# Public policies and conservation plans of historic urban landscapes under the sustainable heritage tourism milieu: discussions on the equilibrium model on Kulangsu Island, UNESCO World Heritage site

**DOI:** 10.1186/s43238-023-00086-0

**Published:** 2023-05-05

**Authors:** Long Zhao, Yuan Li, Na Zhang, Zhenxin Zhang

**Affiliations:** 1 School of Architecture and Civil Engineering, Xiamen University, No. 422, Siming South Road, Xiamen, 361005 Fujian China; 2 Xiamen Key Laboratory of Integrated Application of Intelligent Technology for Architectural Heritage Protection, Xiamen, 361005 China; 3 Department of Architecture, Xiamen University, No. 422, Siming South Road, Xiamen, 361005 Fujian China; 4 School of Architecture Urban Planning Construction Engineering, Polytechnic University of Milan, Piazza Carlo D’Arco, 3, 46100 Mantua, Lombardia Italy

**Keywords:** sustainable heritage tourism, historic urban landscapes, equilibrium model, public policies, conservation plans, Kulangsu Island

## Abstract

**Purpose:**

The tensions and threats in historic urban landscapes brought about by heritage tourism are still regional, global, general, and dynamic issues. For Kulangsu, there is an obvious problem in the connection between the current conservation plan and public policy. To a large extent, public policy cannot effectively, specifically, and flexibly respond to the dynamic problems in the implementation of the conservation plan, which seems insufficient concerning the effect of these conservation plans and public policies on promoting the adaptive reuse and sustainable tourism of the historic urban in Kulangsu heritage sites. Thus, giving more consideration to the combination of public policies and conservation plans of historic urban landscapes under the heritage tourism milieu, ensuring a balanced, sustainable, and integrated development pattern still calls for new discussions in achieving good performance of sustainable heritage tourism. This study conceptually discusses the equilibrium model of historic urban landscapes with a range of strategies under a sustainable heritage tourism background and responds to the synthetic contradiction of the imbalances among public policy, conservation plans, and development practices.

**Design/methodology/approach:**

The study is based on a range of prepared desktop studies (public policy studies, conservation plans), field surveys, participant observations, and randomised interviews to respond to the insufficiency of the current heritage practices.

**Findings:**

This study discusses the equilibrium model of sustainable heritage tourism at heritage sites. It takes Kulangsu Island, a UNESCO World Heritage site in Southeast China, as an example to discuss the equilibrium model, which encompasses a convergent parallel framework and three dimensions concerning heritage management and policymaking. The equilibrium model of historic urban landscapes is a dynamic framework that integrates social, economic, environmental, and cultural concerns into a holistic collaborative framework under a sustainable heritage tourism background.

**Originality/value:**

In line with the requirements of the Historic Urban Landscape (HUL) approach and general principles in support of sustainable urban heritage management promoted by UNESCO and ICOMOS, the study points out the peculiarities and potential of the equilibrium mode in solving the current challenges of historic urban landscapes for sustainable heritage tourism. Finding ways of linking policymaking, conservation, development, heritage tourism, and different interest groups to a holistic framework can stimulate effective means and management mechanisms for the complicated and changeable issues of sustainable heritage tourism.

## Introduction

The rapid development of heritage tourism in recent decades has benefited from the difficult work of maintenance of the social, cultural, and historical environment of heritage sites. Meanwhile, international and multilateral agencies and domestic tourism policies, strategic planning, and operation have also provided sufficient support for the realisation of the goal of tourism development at heritage sites. However, as a double-edged sword of heritage tourism, the relationships among historic urban landscapes, conservation, heritage tourism, and stakeholders seem difficult to maximise in balancing the interests of all parties, and there are still many queries raised and pressures regarding the conservation of the historic urban landscape authenticity, integrity, and sustainability attributes in heritage tourism projects. The result is that current practice has led to a benefit-centred development model and market-oriented policymaking.

Many heritage items are endangered by rapid economic development resulting from the government’s economically driven policy in China (Chan and Ma 2004). Is there a mechanism to coordinate or mitigate the conflict between conservation and development? The arrival of the era of sustainable development brings new challenges and standards to the historic urban landscape in historic cities, and public policies and conservation plans increasingly became the main actions for achieving the goal. In retrospect, the dissemination of sustainable development was facilitated by a series of international documents after the 1980s, which had a comprehensive impact on the formulation and implementation of national public policies, conservation programs, and tourism development agendas. Documents such as *The Venice Charter for the Conservation and Restoration of Monuments and Sites* (1964), *The Florence Charter* (1982), *Convention for the Protection of the Architectural Heritage of Europe* (1985), *Charter for the Conservation of Historic Towns and Urban Areas* (1987), *The Nara Document on Authenticity* (1994), and *Charter of Cultural Tourism* (1996) incorporate the concept of sustainable development in varying facets and undoubtedly generate extensive and widespread impacts on state parties’ policy formulation and implementation. In this light, these documents not only spread the concept and principles around the world but also offer a standard sample for state parties to formulate and update their domestic public policies and series of conservation plans.

The tensions and threats in historic urban landscapes brought about by heritage tourism are still regional, global, general, and dynamic issues. UNESCO adopted the *Recommendation on the Historic Urban Landscape* on 10 November 2011, which emphasises the importance of the historic structure formed by the profound influence and interweaving of natural and cultural elements of a wide range of social, economic, and historical backgrounds (UNESCO 2011; Li et al. 2022). For Kulangsu, there is an obvious problem in the connection between the current conservation plan and public policy. To a large extent, public policy cannot effectively, specifically, and flexibly respond to the dynamic problems in the implementation of the conservation plan, which seems insufficient concerning the effect of these conservation plans and public policies on promoting the adaptive reuse and sustainable tourism of historic urban landscapes in Kulangsu heritage sites. Thus, giving more consideration to the sustainability of historic urban landscapes under the heritage tourism milieu and ensuring a balanced, sustainable, and integrated development pattern still calls for new discussions in achieving good performance. This study conceptually discusses the equilibrium model of historic urban landscapes under a sustainable heritage tourism background and responds to the synthetic contradiction of the imbalances between conservation and development when public policies and conservation plans work in practice. This study takes Kulangsu Island, a UNESCO World Heritage site in Southeast China, as an example to discuss the equilibrium model, which encompasses a convergent parallel framework and three dimensions concerning heritage management and policymaking. The equilibrium model of the Historic Urban Landscape (HUL) approach is a dynamic framework that integrates social, economic, environmental, and cultural concerns into a holistic collaborative framework under a sustainable heritage tourism milieu.

## Sustainable heritage tourism and public policy

Tourism has long been used as a development tool to create jobs, generate tax revenues, stimulate entrepreneurial activity, improve infrastructure and recreational opportunities, promote urban regeneration, empower residents, and improve a destination’s quality of life overall (Wall and Mathieson 2006; Kim et al. 2013; Lak et al. 2020). Sustainable cultural tourism offers a new perspective, as it places cultural heritage and local communities at the centre of decision-making processes (European Commission 2020). Scholars define sustainability in heritage tourism as a four-dimensional concept, namely, the economic viability of the entire operation, ecological and cultural sustainability of the heritage site and its surrounding environment, institutional consolidation of transparent institutional structure, and maintenance of a fair and equitable distribution of costs and benefits (Li and Hunter 2015). In the tourism context, ‘sustainable heritage tourism’ emphasises the contemporary need to balance the conservation of built heritage, heritage tourism policymakers, tourism profits, and stakeholders’ interests with the overall goal of a conservation plan and long-term development agenda.

Public policies are actions carried out by public authorities that aim at the common good and meet the needs of society, being oriented towards meeting public interests (Baptista et al. 2019). The broad meaning of public policy includes measures such as ordinances, laws, plans, guidelines, and strategies for action. that are adopted to manage the urban landscape (Sun and Wang 2000). Policies will need to be implemented to allow the area to remain alive and prosperous but at the same time to ensure that any new development is in accord with the area’s special architecture and historic interests (Municipal Council of Penang 2005). In this paper, the concept of ‘public policy’ is under the research frame of conservation and development, which includes policy making, policy execution, supervision, and feedback under the background of regional characteristics and contexts.

## Literature review

### Heritage conservation and tourism based on sustainability concepts

Concerning heritage conservation, heritage tourism, and sustainable development, a range of scholars have carried out various explorations, such as the multicriteria decision-making model (MCDM) for urban built heritage conservation (Yau 2009), hybrid-modified multiple attribute decision-making (MADM) models for the improvement of heritage tourism performance (Peng and Tzeng 2019), the modality of critical ethnography regarding tourism, heritage, and cultural performance for heritage tourism (Santa and Tiatco 2019), and models of heritage tourism sustainable planning (Mrda and Caric 2019). These models are based on evaluation, theoretical exploration and planning practice to explore strategies and methods for sustainability considerations.

### The combination of public policy, intervention mode, and tourism in heritage sites

**Fig. 1:**
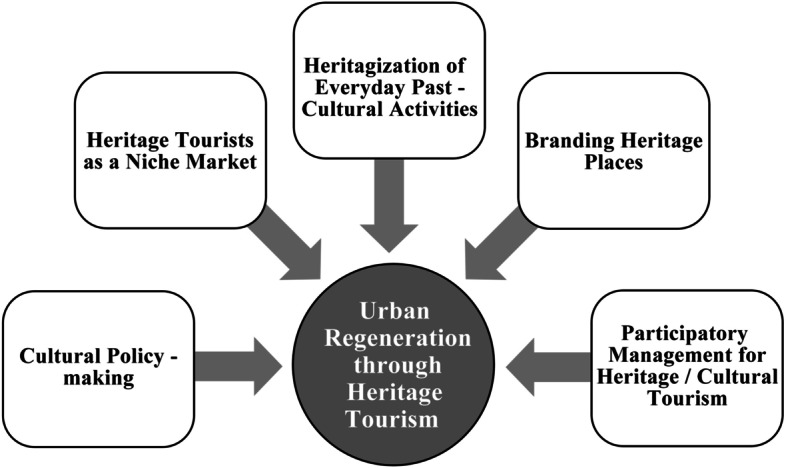
Conceptual framework of heritage tourism as a tool for urban regeneration (Source: the author, based on Lak, Gheitasi, Timothy 2020)

Public policy is considered strong when it solves problems efficiently and effectively, serves justice, supports governmental institutions and policies, and encourages active citizenship (Sun and Wang 2000). J. Wang (2019) discussed an assemblage approach in the conservation of large-scale heritage sites, including the apparatus of intervention and establishing new power relations. Baptista et al. (2019) discussed the connection between tourism and public policies, leading to the vision of sustainability for economic development and a tool for building a more prosperous community. Lak et al. (2020) stated that heritage can be utilised in contemporary situations and mobilised for a variety of present objectives and public policy purposes. They discussed the significant role of heritage tourism as a tool for urban regeneration and developed a conceptual framework for urban regeneration through heritage tourism (Fig. 1).

### Kulangsu island and its heritage assets

**Fig. 2:**
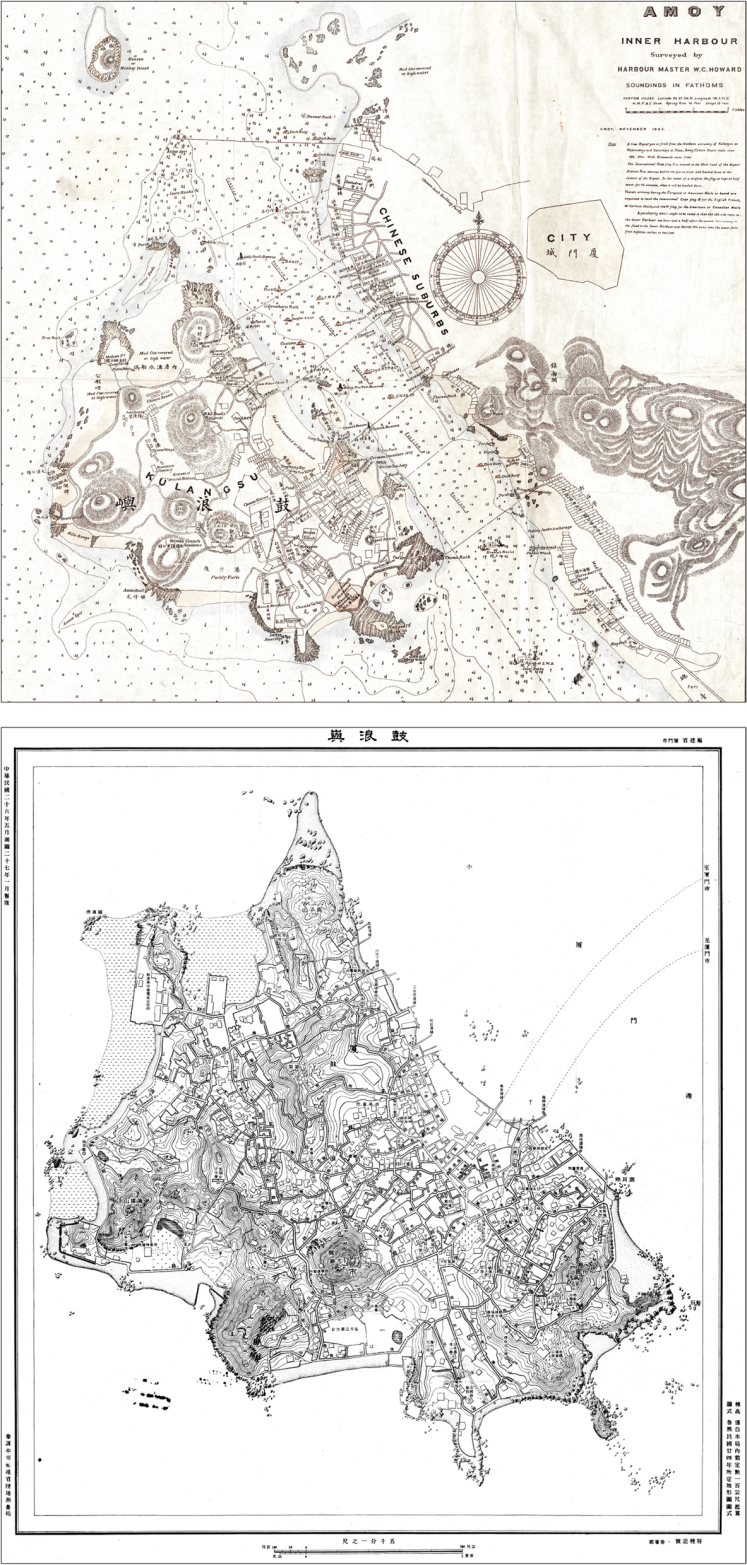
Kulangsu Island in 1892 (above) and 1937 (below) (Source: the Management Committee of Xiamen Kulangsu Island-Wanshishan Scenic Area, China)

**Fig. 3:**
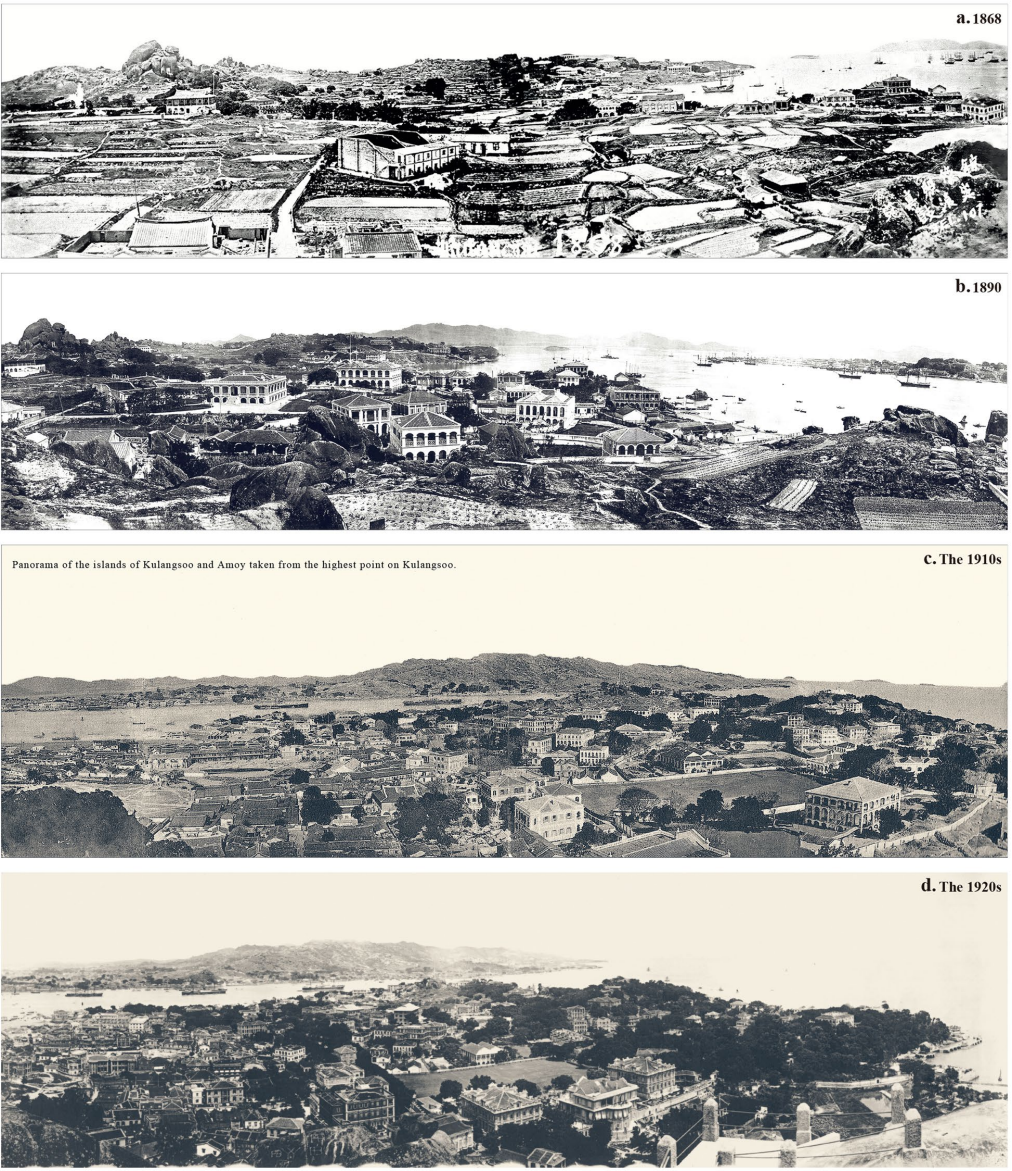
Historic urban landscapes of Kulangsu Island in 1868, 1890, the 1910s, and 1920s (Source: the Management Committee of Xiamen Kulangsu Island-Wanshishan Scenic Area, China)

Kulangsu Island is located in southeastern China along the Taiwan Strait. The island is an irregular oval approximately 1,800 m long and 1,000 m wide, with an area of only 1.78 square kilometres (Fig. 2). With the opening of a commercial port at Xiamen in 1843 and the establishment of Kulangsu Island as an international settlement in 1903, this island off the southern coast of the Chinese empire suddenly became an important window for Sino-foreign exchanges (UNESCO 2017). From the middle of the 19th century to the middle of the 20th century (Fig. 3), with the opening of Xiamen, Kulangsu Island has become an important window for cultural exchanges and integration between China and foreign countries, from fishing village islands to international communities to historical communities after the reform and opening up of China, and was finally listed as a world cultural heritage site in 2017. Consulates, churches, hospitals, schools, police stations, etc., built by those foreign communities explain the predominantly Victorian-era style architecture that can still be seen throughout Kulangsu Island. Hence, Kulangsu Island has undergone tremendous changes and has experienced rapid growth since the advent of the colonial era. The island can be treated as a typical case to explore the evolving urbanscapes and the integration of different cultures, which together yield unique characteristics. Both the built heritage and the natural sceneries of Kulangsu Island are unique evidence of history and culture, and the evolution of its community space has significant research value.

In the 1920s, a large number of overseas Chinese returned from Nanyang, and they built schools, churches, hospitals, banks, post and telecommunications offices, villas, private gardens, and all kinds of houses on Kulangsu Island, making it a historical international community with the integration of Western, Nanyang, and Southern Fujian cultures. According to statistics, during the 1920s and 1930s alone, returning overseas immigrants built 1,014 buildings on Kulangsu Island. According to other statistics, among the 970 construction licences issued by the Municipal Council between 1924 and 1936, 75 percent belonged to returning overseas immigrants and their dependants (Dai 2018). The architectural ensemble and urban fabric of Kulangsu Island are mixed with southeast Asian and European architectural and cultural characteristics. There is a mixture of different architectural styles, including the traditional Southern Fujian style, Western classical revival style, and veranda colonial style. The most exceptional testimony of the fusion of various stylistic influences is a new architectural movement, the Amoy Deco Style, which is a synthesis of the Modernist style of the early 20th century and Art Deco (UNESCO 2017).

In this study, many sessions of the investigation were conducted using field surveys, participant observation, randomised interviews, and literature analysis. Randomised interviews were conducted among pedestrians, covering the period of November 2020 to August 2022, with a total of 112 interviewees, mostly locals, shopkeepers conducting business on Kulangsu Island, a small number of visitors, and cultural preservationists. Due to the COVID-19 epidemic, locals and tourists were significantly impacted during this time, fewer people were on the streets, and most businesses were closed.

### Future agendas and current realities concerning heritage tourism of Kulangsu’s historic urban landscape for making historic cities

**Fig. 4:**
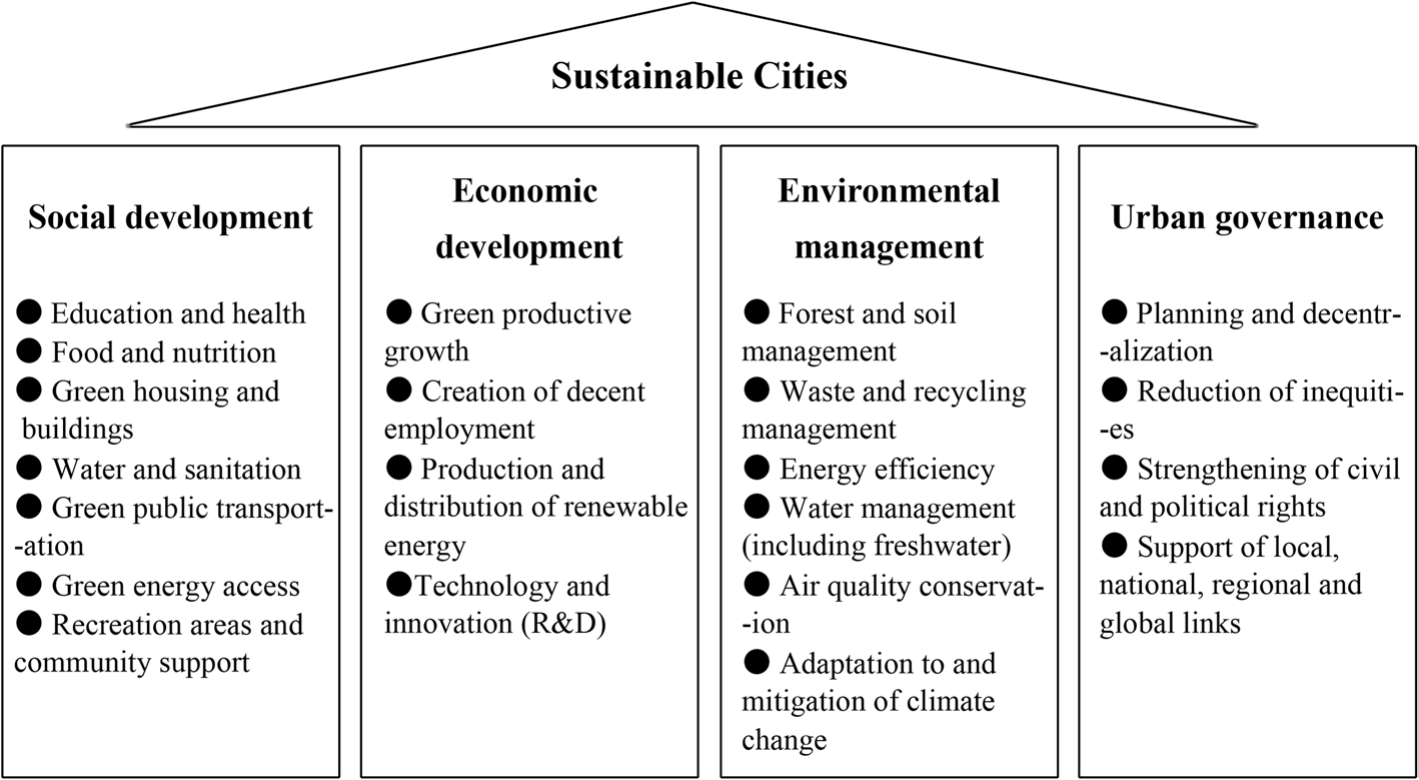
Pillars for achieving sustainable cities (Source: Konbr 2019)

Changing historic urban landscapes from vulnerability to sustainability is not easy in the foreseeable future. As with sustainable cities (Fig. 4), a multifaceted and comprehensive framework determines the practical effects of public policy and master planning responses, while historic urban landscapes are also faced with unpredictable obstacles in a dynamic process. Generally, the historic cities consist of general historic urban landscapes, the cluster of houses, settlements, villages, communities, places of worship, local life-supporting systems, and settings on the island that evolve and develop through long-term human habitation. The significance of their historic urban landscapes is rooted in cultural diversity, collective memories, architectural patterns, ethnic symbols, building materials, social life and livelihood, ideology and so on, which are integrated into the physical configuration. These factors intertwined within the cosmic order and functional order along the historical timeline, layer by layer, which was equipped with significant attributes for developing historic cities and heritage tourism in Xiamen City.

**Fig. 5:**
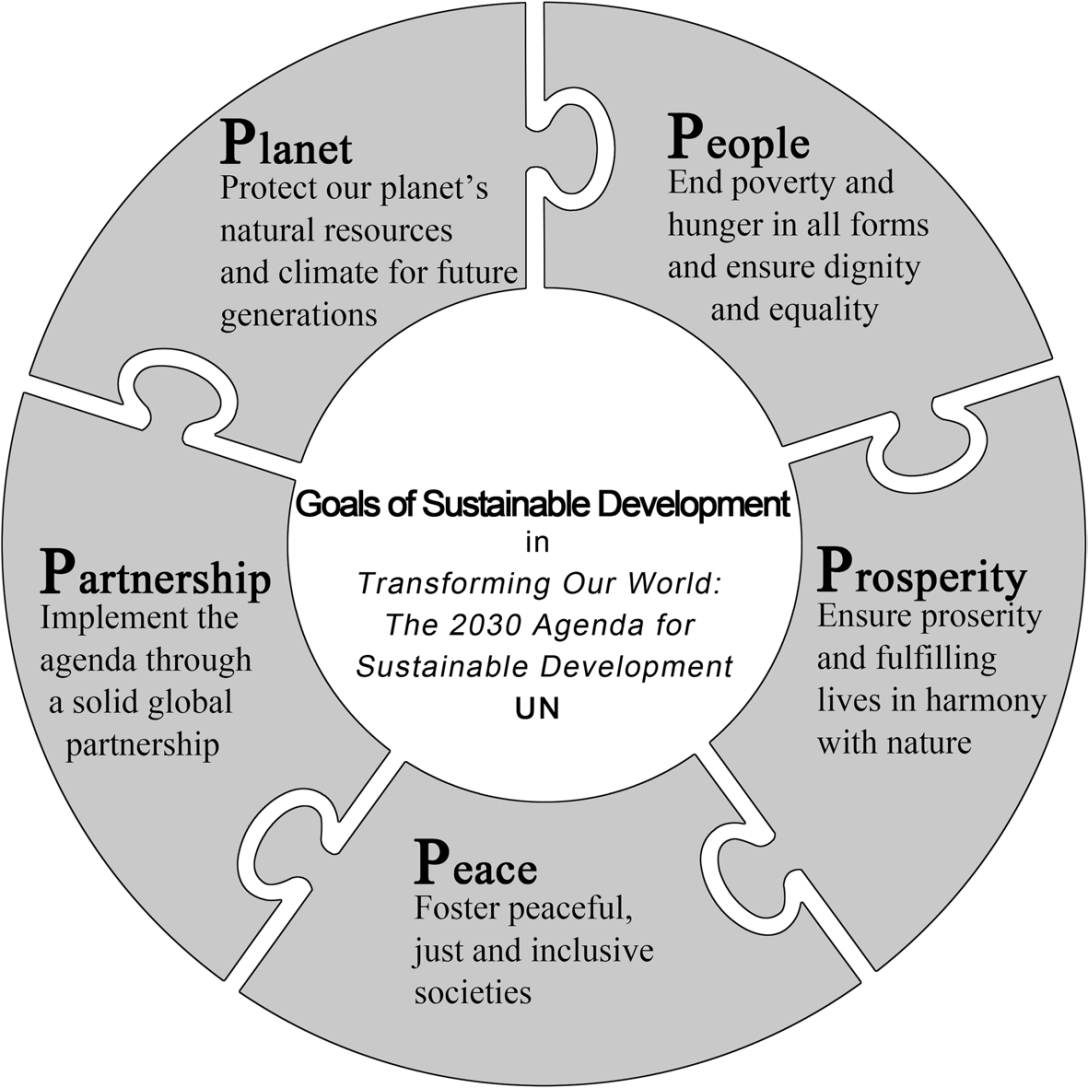
Goals of sustainable development in resolution to transmit the Agenda 2030, UN (Source: the author, based on Suliman 2015)

The UN’s *Transforming Our World: The 2030 Agenda* for Sustainable Development provides 17 sustainable development goals (Fig. 5) and 169 targets to respond to the vision of sustainability through integrated and indivisible and balanced three dimensions of sustainable development, the economic, social, and environmental, and it seeks to combat growing problems and build sustainable, cultural, harmonious, and inclusive urban environment. For the concept of circles of sustainability, a method for understanding and assessing sustainability (Paul et al. 2014), a series of Sustainable Development Goals (SDG) indicators are formulated by transferring a conceptual definition to clear dimensions for the long-term planning process and holistic strategy-making. In regard to heritage tourism on Kulangsu Island, fewer discussions have been conducted to take actual actions to perform the SDG indicators, and more notably, future agendas and current realities concerning sustainable heritage tourism are unclear.

For the historic urban landscapes on Kulangsu Island, first, we consider the condition of physical capital, which is represented by its physical configuration such as geographical features, urban infrastructure, old building clusters, landmarks, fabrics, morphology, vegetation, and overall townscapes, reflects the sustainable consideration of heritage tourism; and second, we consider the cultural and socioeconomic values that they signify and the role that these perform in defining cultural diversity, widespread consensus, sense of place, sense of history, place spirit, community belonging, business patterns, and social cohesion. As we have seen, in the process of policy formulation and planning-based practice for the conservation of historic urban landscapes, there are continuing concerns over economic sustainability in most historic cities, mainly focusing on income distribution, employment rate, new business types, equality of competitive opportunities, inclusive and green economic growth model, investment in heritage education and services, regional economic integration and interconnectivity, and other economic byproducts associated with urban marketing and urban branding, as well as cultural exports through heritage tourism as a tool.

Although there are a series of public policies and conservation plans regarding cultural heritage on Kulangsu Island from the municipal level to the state level, the content related to the sustainable heritage tourism development of the historic urban landscapes is limited. Institutionally, public policies and conservation plans are midway between the conservation and sustainable development of historic urban landscapes. They are fundamental in achieving a sustainable approach to historic urban landscapes and preventing them from being overexploited and degraded. The wise use of heritage assets and effective management will contribute to reversing cultural loss, urban aesthetic degradation, environmental deterioration, etc. For historic urban landscapes on Kulangsu Island, it is recognised that the realistic benefits of sustainability always refer to strategies to combat current issues at the local and regional levels, to promote overall historic urban landscapes to a high level, and to enlarge the scope of social implications, practical implications, and theoretical implications with cross-disciplinary collaborations.

The sustainable heritage tourism of historic urban landscapes on Kulangsu Island is indeed a new direction for branding Kulangsu Island as a multicultural living heritage city. It is a practice that is bilateral, involves multilateral co-operation, and is sustainability-oriented in its processes. Comprehensive and integrated methods concerning sustainable concepts accompanying a multidimensional system are often a general solution by heritage administrators predominantly adopted at the local, regional, and even state level (at the territorial scale), and there are numerous cases of more scientific and reliable examinations of this combination. Accompanying this, public policy and conservation plans play a vital role as well as serving as synergistic functions in pursuing sustainable development goals. In this sense, in the historic urban landscape, whether large or small, these two parts should always be intertwined and collaborate with the follow-up sustainable development actions directly or indirectly.

## Equilibrium model: Convergent parallel framework and three facets for sustainable heritage tourism in the historic urban landscapes of Kulangsu Island

### Convergent parallel framework between conservation and development

**Fig. 6:**
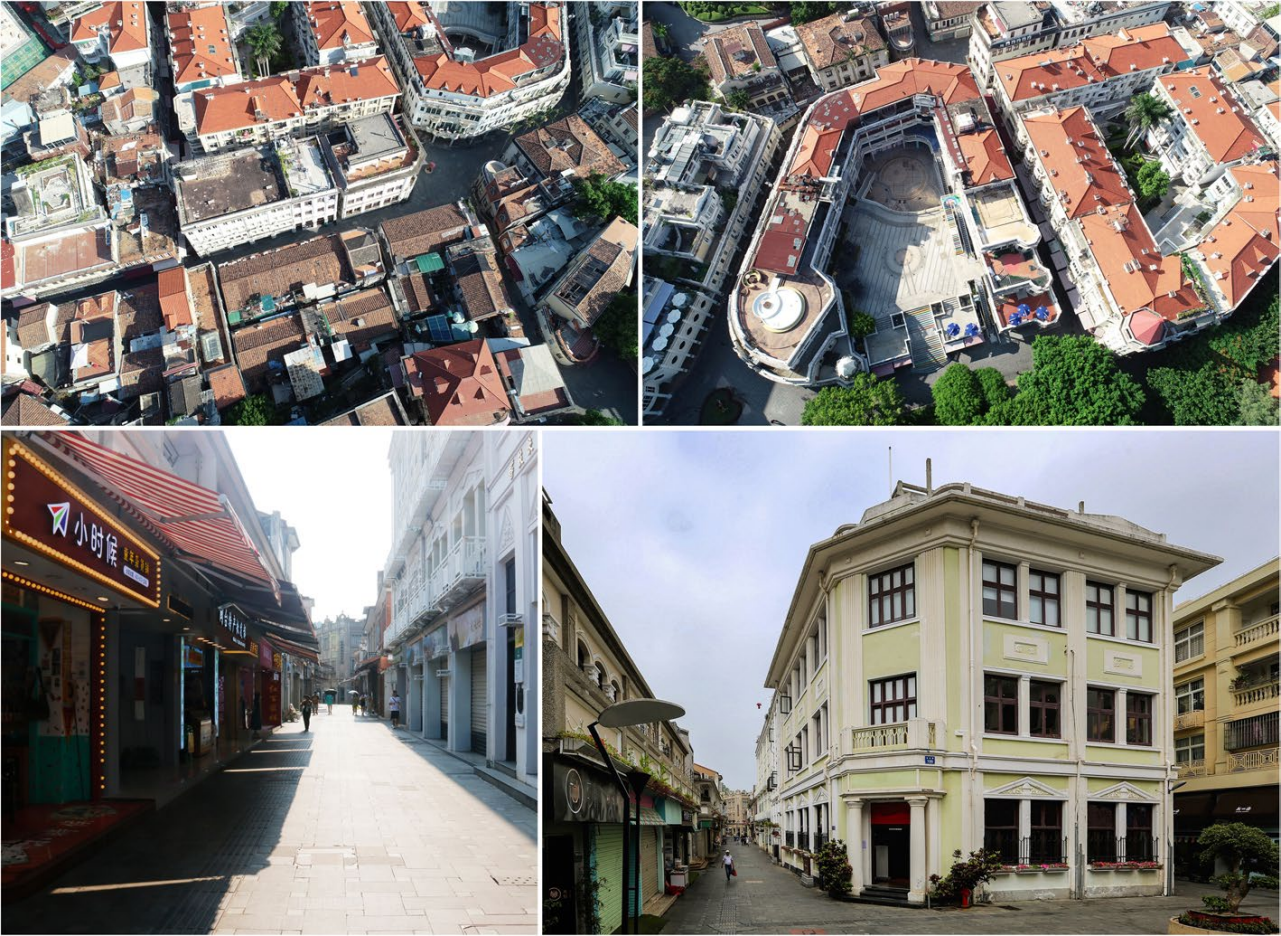
The historic Commercial street of Longtou Road on Kulangsu Island: like a ghost town during the COVID-19 epidemic, 2022 (Source: Mengsheng Yang)

On Kulangsu Island, sustainable heritage tourism development is never limited to one point of view. Before talking about any development models on Kulangsu Island, one question needs to be considered further: what is the big picture of Kulangsu Island’s sustainable heritage tourism? The picture of Kulangsu Island’s cultural diversity that is represented by the UNESCO World Heritage site does not comprehensively or accurately reflect the local diverse history. At the heart of the issue are the questions what constitutes Kulangsu Island’s historic urban landscapes, and why are they of value? How do we reconcile authenticity, integrity, and modernity without discarding anything or everything historic as we transition into the modern era? How do we balance conservation work and sustainable heritage tourism development? Essentially, the solutions depend on the needs of an equilibrium model within the short- and long-term public policies and conservation plans, especially for the recovery of holistic conservation and tourism development after the COVID-19 epidemic (Fig. 6).

The focus on the promising development vision of historic urban landscapes on Kulangsu Island undergoes continuous discussion for dealing with new issues and urban demands. The sustainable development of a historic city and the sustainability of historic urban landscapes are essentially about negotiating the transition from past to future in coordination and dynamic mechanisms to ensure the transfer of maximum significance. This is not only the most convincing argument for conservation but also renders it a serious responsibility for long-term development. The concept of combining conservation with the sustainability of urban assets at the global through local scale is not new, but the outcomes are different in both developing countries and developed countries. Indeed, it seems that the bond between conservation and sustainability is complicated, difficult to control, and affected by multiple factors. While people are passionately interested in conserving historical remains, retaining and interpreting the significance of heritage in all its difficult complexity, and promoting heritage as a new economic driving power on Kulangsu Island, the existence of multiple conflicting problems suggest that heritage administrators adjust their public policies and conservation plans. A major challenge lies ahead here. On the one hand, identifying the major changes and issues in the historic urban landscapes and public policies/plans should be high on the agenda, but on the other hand, theorising these changes and reformulating existing policies, plans, or strategies in a way that would permit illuminating and exploring the dilemma or contradiction of new situations is also urgently needed. This concept provides the link between the preservation of the past for its intrinsic value and as a resource for sustainable development.

**Fig. 7:**
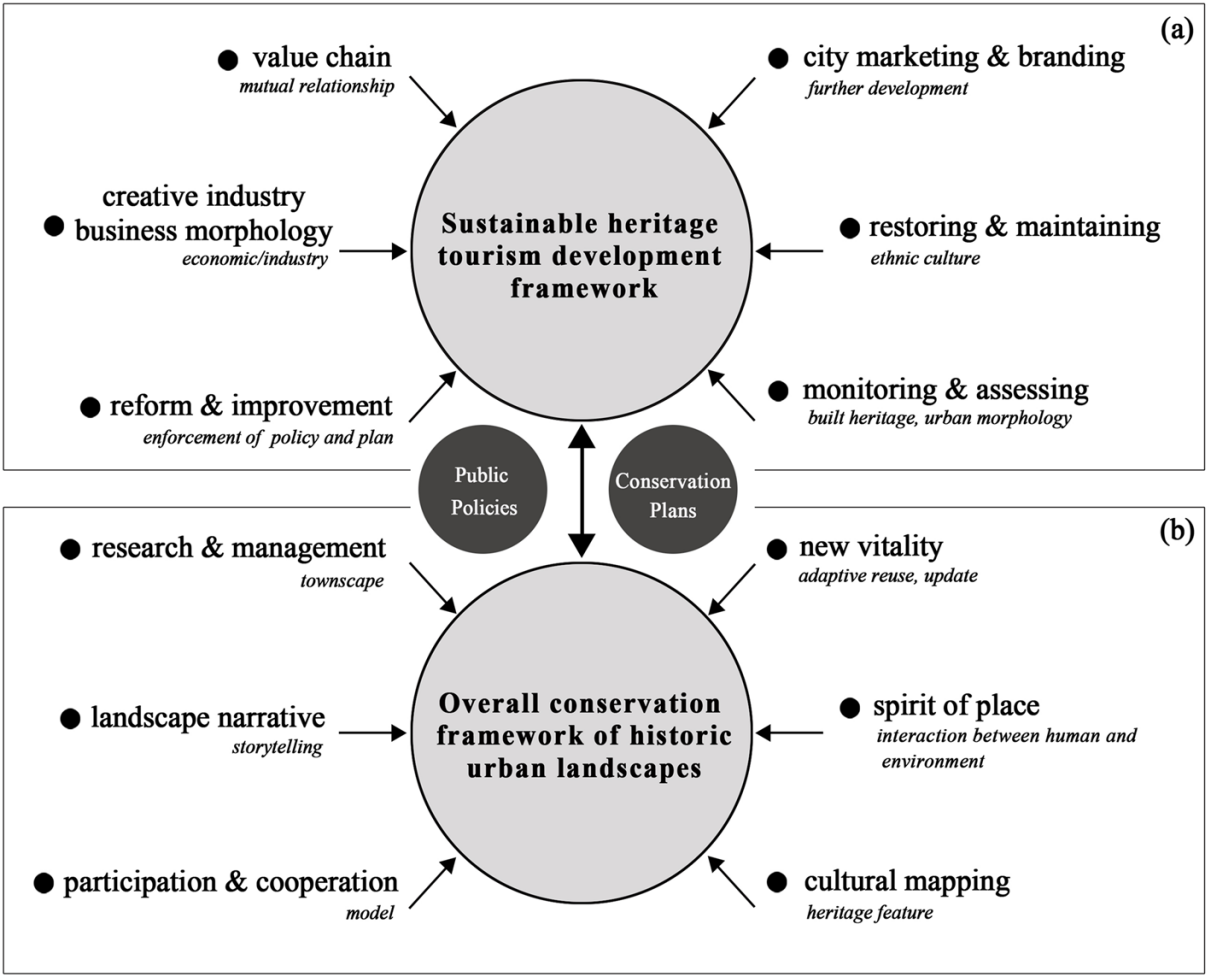
Convergent parallel framework for sustainable heritage tourism (Source: the author)

In general, good strategies should keep pace with reality for remaking the urban image, conserving historic urban landscapes, encouraging cooperation and equality, and enforcing these policies effectively. The development and conservation of historic urban landscapes on Kulangsu Island, therefore, is a process requiring the understanding and appreciation of historic remains with a long-term sustainable vision, not just limited to practical economic interests or only intermittent conservation practices. Here, this study divides the convergent parallel framework into two parts (Fig. 7): a sustainable heritage tourism development framework for incentivising tourism growth and an overall historic urban landscapes conservation framework for maintaining heritage value, which contributes to establishing its meaning. A long-term conceptual mechanism is discussed in the following two parts:

#### Sustainable heritage tourism development framework

The nature of sustainability incorporates social, cultural, and economic dimensions and demonstrates strong interdependencies between the environment and people (Manzi et al. 2010; Ardakani and Oloonabadi 2011). For sustainable heritage tourism development, sustainability is first reflected in the condition of physical capital, which is represented by its physical configuration, such as geographical features, urban infrastructure, old buildings, landmarks, fabrics, morphology, vegetation, and townscapes; and second, the sociocultural values that it signifies and the role that it performs in defining cultural diversity, sense of place, sense of history, local spirit, community belonging, business patterns, and social cohesion.

The *Recommendation on the Historic Urban Landscape* (UNESCO 2011) contributes to increasing the impacts of the conservation of cultural heritage in terms of creativity, resilience, and sustainability and is very useful to stress the integration of heritage conservation into city planning, breaking the prescriptive approach of conservation charters (Fusco Girard 2013). According to the historic urban landscape approach, pursuing sustainable townscape development should respect local communities in their quest for development and adaptation while retaining the characteristics and values linked to their history and collective memory and the heritage environment. This approach aims to re-establish the connection between the management of the historic environment, contemporary urban development, and the geological context (Margottini and Spizzichino 2015). Furthermore, this constant adaptation to human needs can actively contribute to maintaining the continuum among the past, present, and future life of our communities (ICOMOS 1996).

According to the People-Centred Approach (ICCROM 2015), cultural heritage has been created by people and for people. Taking a people-centred approach in townscape studies is not merely a suggestion for increasing participation within a management system but also addresses a core component of heritage management—the people who are connected to heritage—and ensuring that it is an integral element of conserving that heritage. It retains a focus on its more qualitative and humanistic spectrum. In this light, the role of ethnic culture and urban characteristics is highlighted, and the urban image are improved.

#### Overall historic urban landscape conservation framework

Earlier studies of colonial/postcolonial cities, cultural exchange/immigration, history/politics, architecture/context, and trade/society (principally in English and Chinese) produced during the independence of Malaya form a comprehensive view of Kulangsu Island. In this context, Kulangsu Island is a historic site, and continuous studies should be carried out to consider factors such as landscape preferences, soundscapes, colorscape planning, overall visual assessment, city representation, heritage tourism, city marketing/branding, environmental impact assessment, and creative economy. Historic urban landscapes mirror the character of a specific ethnic group and absorb new energy in the evolution process. Following this train of thought, the management of the overall townscape should start and focus on the ethnic group or local community itself, the history/contexts, ways of life (cuisine, activities, and behaviours), emotional/spiritual activities (including ideology, thinking, and religion such as place attachment/rootedness on Kulangsu Island), language/literature, etc.

In doing so, first, we introduce a constructive dialogue and negotiation mechanism to answer the critical questions at the outset between heritage administrators and local dwellers (identify problems and solve them in time) in order to consider the voices from the grassroots. As Ken Taylor (2015) argues, it is fundamentally important to listen to community voices and learn how to communicate findings to planners, politicians, and developers who will be influential in making land-use policy decisions. He points out that a model for listening to the community voices of ordinary people in a historic urban setting is found in the work of Lim Huck Chin and Fernando Jorge in Malacca: ‘We began by listening to the voices of ordinary Malaccans. We listened to the city’s streets, as we searched out hidden corners and abandoned alleyways. Listened to houses and temples, ruins and cemeteries… And we heard them speak’ (Lim and Jorge 2006). The importance of local involvement in the processes and decision-making related to cultural landscapes—from identification to the description of their values to the nomination, implementation, educational role, and long-term outcomes—is crucial to their sustainability (Nora and Susan 2000).

Second, HUL management on Kulangsu Island cooperates with advisory bodies and statutory bodies. Previous cases demonstrated that all problems related to preservation and restoration can be solved through cross-departmental and ministerial collaboration. Heritage administrators should investigate the conservation practice and efficiency of public policies/plans regarding the overall townscape with the help of advisory bodies and statutory bodies. Although these professionals are not directly involved in any business activities in old shophouses, they can help communities increase their capacity for business transformation and knowledge of conservation. Citizens supported by professionals are empowered to promote the development and systematisation of heritage preservation (Nyseth and Sognnæs 2013; Lee and Shih 2018).

Third, cooperation and win‒win mechanisms should be constructed between government agencies and stakeholders (landlords, residents, business owners, and local business groups). On the one hand, the rights of local communities should be recognised, and on the other hand, these agencies should work with the widest possible participation of stakeholders. The overall management of historic urban landscapes is a multidisciplinary field that involves inputs from various professionals, including politicians, architects, planners, engineers, historians, archaeologists, environmentalists, and other experts. Heritage administrators can work effectively with these professionals and NGOs.

### Three dimensions: Social, economic, and public policies & conservation plans

#### Social dimension: Reconnecting cultural diversity and stakeholders’ engagement in sustainable heritage tourism development

Broadly speaking, social sustainability refers to the compatible and harmonious development of society, which has a changing role according to the development of contemporary society. In the field of heritage conservation, the social dimension of sustainable heritage tourism development emphasises the need to improve the quality of life for all citizens by making the location a better place to live and work and empowering community action, social equity, inclusiveness, and ownership, which is consistent with the concerns of built heritage conservation as part of sustainable development (Delafons 1997; Townshend and Pendlebury 1999; Strange and Whitney 2003; Tweed and Sutherland 2007; Yung and Chan 2012). Social sustainability is a basis for pursuing good performance of the historic urban landscapes and SDGs in the long term. Given this context, the discussions of the social sustainability of the HUL Recommendation focus more on cultural diversity and community engagement through a range of ongoing collaborations and interactions among different clusters in the heritage site or a historic city, which results in social integration, equity, social justice, balance, solidarity, inclusiveness, acculturation, opportunities, improvements in quality of life, etc.

One of the central arguments of the study, as mentioned before, is demonstrating the socially progressive potential of the HUL Recommendation to stimulate conservation and sustainability through community-led collaboratives, policy-led planning, and heritage-based place-making to reconnect cultural diversity and community engagements compatible with UNESCO’s SDGs. The findings of street interviews on Kulangsu Island reveal that both the dwellers and stakeholders have a good understanding of the heritage value they live with every day, and that they have a positive outlook towards the, multiculturalism, social identification, acculturation, and competitiveness brought about by cultural diversity in the place. It also shows that local citizens and stakeholders understand their role in facilitating the sustainability of historic urban landscapes, but they want to be respected in the aspect of equitably economic, social, and cultural benefits, as well as their attitude towards the sustainable development of heritage tourism. However, negative feedback was derived mainly about governmental actions facilitating heritage custody and solidarity among local citizens, but it was not confirmed by the locals. Currently, for social sustainability, the public is willing to take action, but there is a clear gap due to the lack of governmental actions.

First, effective community participation and cultural diversity on Kulangsu Island require equity and social justice, which is in line with UNESCO’s SDGs. Generally, cultural diversity and community engagement are based on social equity, which, as some researchers have pointed out, is a crucial component of social sustainability and economic vitality because of the positive impacts of facilitating the optimised allocation of resources and urban development agendas in the long run. On Kulangsu Island, when trying to pursue sustainable goals of to maintain the Outstanding Universal Value (OUV) and to create an urban brand of the multicultural living heritage city, such as other heritage cities, cultural diversity and community engagements are unavoidable topics. Any social exclusion and marginalisation of a community group in the hyper politicised setting can reap short-term benefits but are ineffective for the overall social and cultural development of the country without civic active involvement. Hence, ignoring cultural diversity and community engagements and without the dominance of social equity, locals are left out of the decision-making process in conservation and sustainable development; consequently, these people may not support conservation, and social sustainability is not achieved (Yung and Chan 2012). Direct participation in historic urban landscapes can positively activate social and cultural vitality, social networks, sense of belonging, and cultural communication, and common interests and visions are developed and presented in the HUL Recommendation. Self-efficacy and sense of pride in the multicultural environment are improved. In addition, it increases the possibility of motivation, opportunity, and ability as part of regenerative processes in sustaining the authenticity and integrity of historic urban landscapes in the process of sustainable heritage tourism development.

**Fig. 8:**
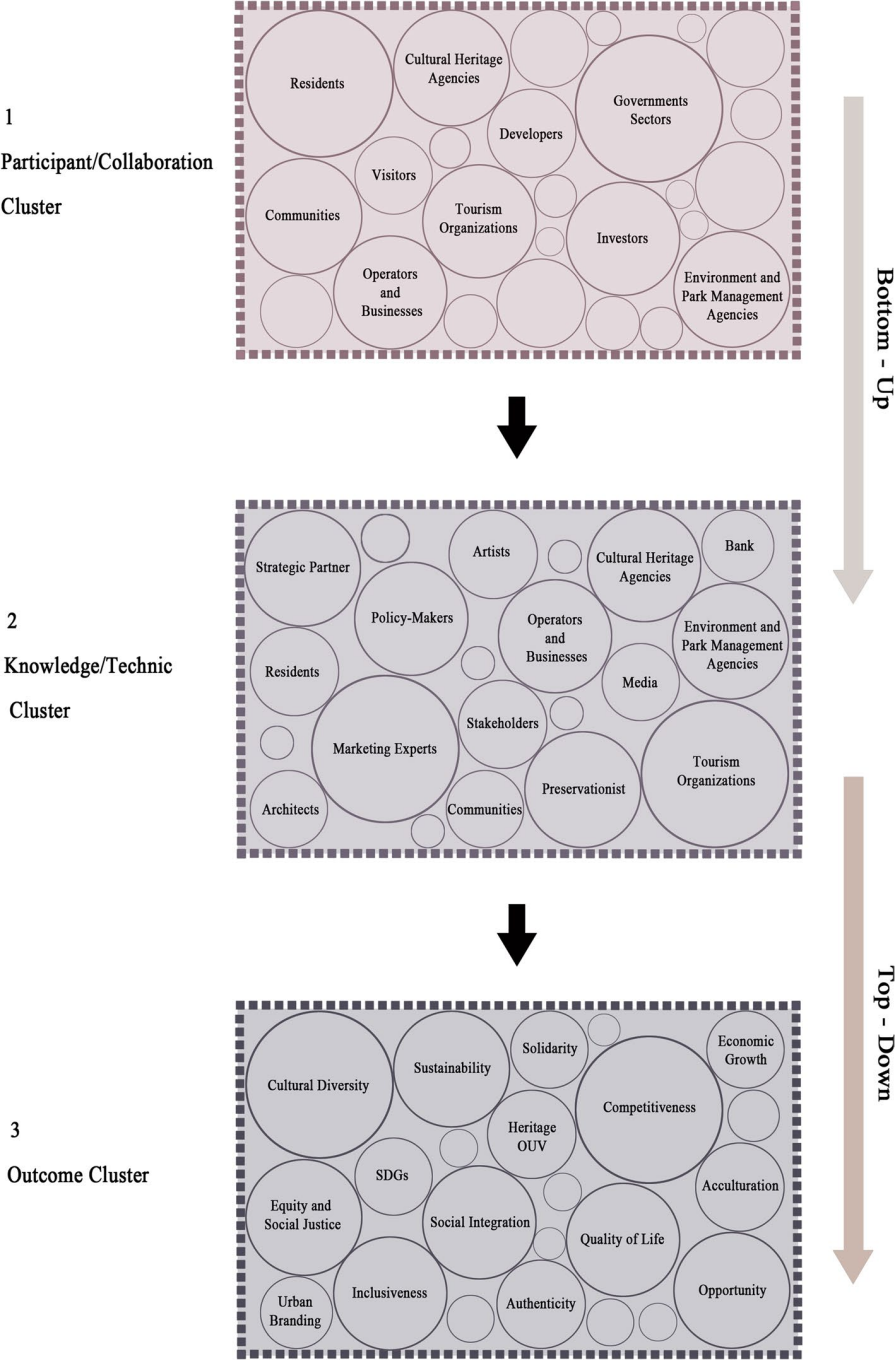
A synergistic working Cluster of potential participants emerges in social sustainability, reflecting strong performance in historic urban landscapes (Source: the author)

Second, channels that allow clear communication are needed for the availability of up-to-date information, decision-making, and financial support. The types of community participation, as some scholars have described, are on a three-tier ladder: coercive participation, induced participation, and spontaneous participation. Compared with coercive participation and induced participation, spontaneous participation highlights the residents’ role in making decisions and participating in the process of both conservation programs and development projects. This view needs to be further explored and put into practice on Kulangsu Island in terms of well-communicated channels. The World Heritage site brought together various communities and cultures to live together within Kulangsu Island, making it truly multicultural and multireligious, with each community free to follow the values and religious beliefs of its respective culture (Rasoolimanesh et al. 2017). The social investigation on Kulangsu Island indicated that many residents are unwilling to discuss political and policy issues. In regard to tourism activities and conservation programs, there are no suitable channels to motivate participation will and enthusiasm. However, residents are eager to participate in conservation programs and tourism development projects based on a win‒win strategy because they have resources that legislation does not have. Thus, as shown in Fig. 8, improving public interaction and constructing well-communicated channels within a synergistic working group is feasible to strengthen social sustainability; moreover, high active participation based on effective channels facilitates the combined effects of social, economic, and environmental sustainability.

Third, UNESCO’s international initiatives concerning inclusive cities bring great opportunities for cultural diversity and community participation on Kulangsu Island in terms of social sustainability. Identification, perception, affiliation, acculturation, inclusiveness, acceptance, diversification, and solidarity will make Kulangsu Island a real multicultural living heritage city and an inclusive city. Economic growth is easy, but inclusion is harder, and even in America, it takes a very long time to extend the benefits of growth and engage more people in prosperity. Removing some barriers and handling social issues properly is crucial to providing opportunities for high- and low-income populations, controlling housing prices, maintaining cultural diversity, and creating a nondiscrimination society on the historic urban landscapes of Kulangsu Island. The concept of inclusive cities involves a complex web of multiple spatial, social, and economic factors (The World Bank 2020). Based on this concept, on Kulangsu Island, spatial inclusion requires providing affordable necessities for civic purposes such as housing, water, and sanitation; social inclusion requires guaranteeing equal rights and participation of all, which is highlighted in this study; and economic inclusion requires providing opportunities for residents in creating jobs and sharing the benefits of economic growth. The three dimensions of the inclusive city are tightly intertwined and tend to reinforce each other. Thus, multidimensional interventions as a holistic approach are needed to put into effect multisector solutions, combining ‘preventive’ and ‘curative’ solutions, optimising investment models in conservation programs and development projects, harnessing communities’ potential as drivers of inclusion, strengthening local governments’ capacity for effective political backing, and fostering multi partnerships both internally and externally.

**Fig. 9:**
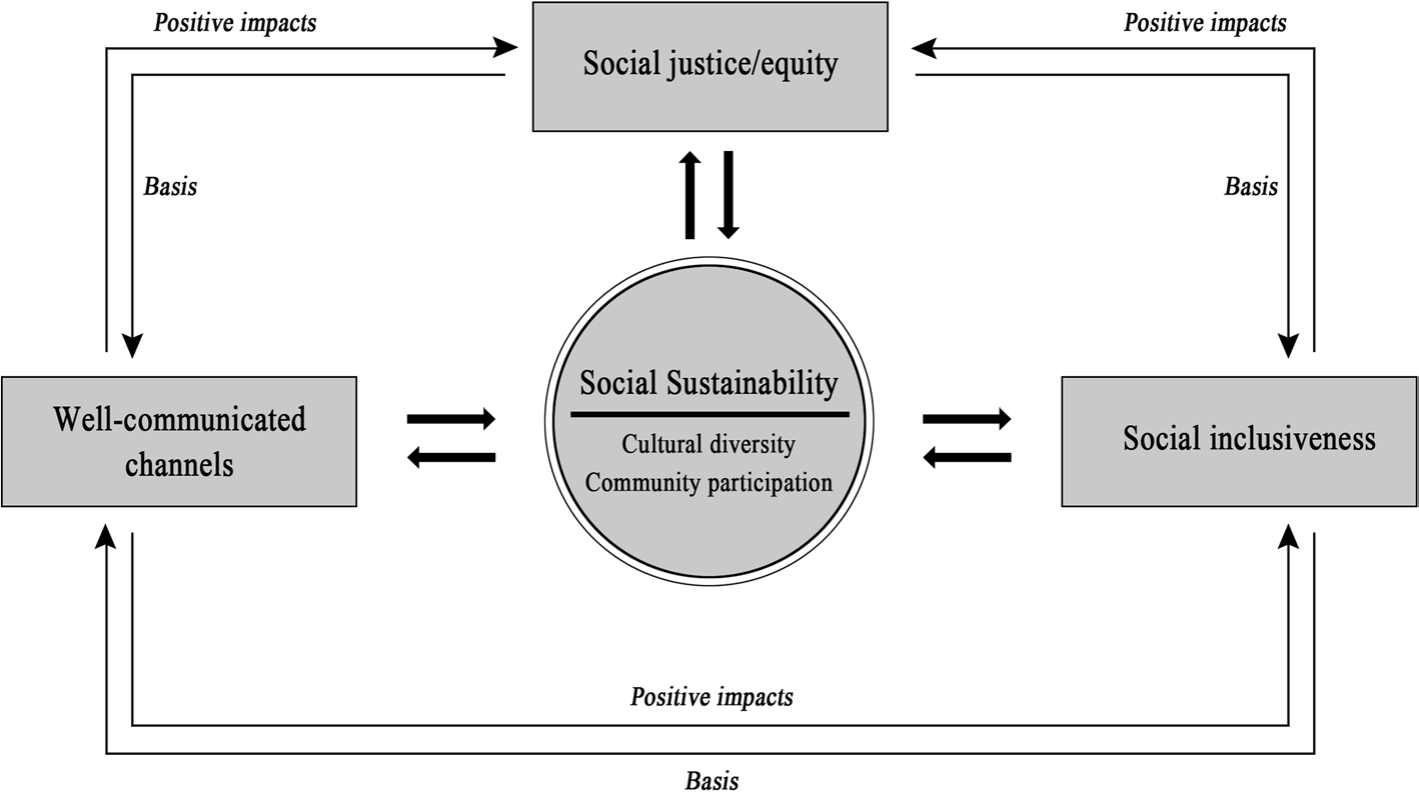
Structure of the internal and external relations of cultural diversity and community participation on Kulangsu Island (Source: the author)

Overall, Fig. 9 presents the structure of the internal and external relations of cultural diversity and community participation in the process of pursuing social sustainability on the historic urban landscapes of Kulangsu Island. The three parts are just the missing parts in social sustainability on the historic urban landscapes of Kulangsu Island, which with their mutual relations produce promotion and restriction influence. Social justice and equity provide a basis for well-communicated channels and social inclusiveness; in contrast, well-communicated channels and social inclusiveness could exert positive impacts on social justice and equity, which contribute to a sustainable and harmonious society in the historic urban landscapes of Kulangsu Island.

#### Economic dimension: The role of the creative industry in heritage tourism and economic vitality

Cultural, social, historical, political, economic, and physical settings all influence the development of heritage items (NSW State Government Authority 2005). To realise a successful sustainable development vision, collaboration and shared strategies are required for the sustainability of historic urban landscapes, the liveability of historic neighbourhoods, the historicity of monuments, the authenticity and integrity of urban heritage assets, and the vitality of the local economy. The contribution of the creative sector to historic tourism and economic vitality is a strategy that cannot be disregarded in terms of economic sustainability.

The discovery of new vitality in sustainable heritage tourism requires adherence to the distinctive conditions of the historic urban landscapes, such as special heritage assets, the level of industrial development, regional religious centres, natural resources, unique geographical locations, and indispensable entities for transregional cooperation. Therefore, to ensure the economic viability of Kulangsu Island, a shift from ‘copy and imitate’ to ‘create and originat’ is needed, along with the development of a distinctive urban branding and marketing proposal.

**Fig. 10:**
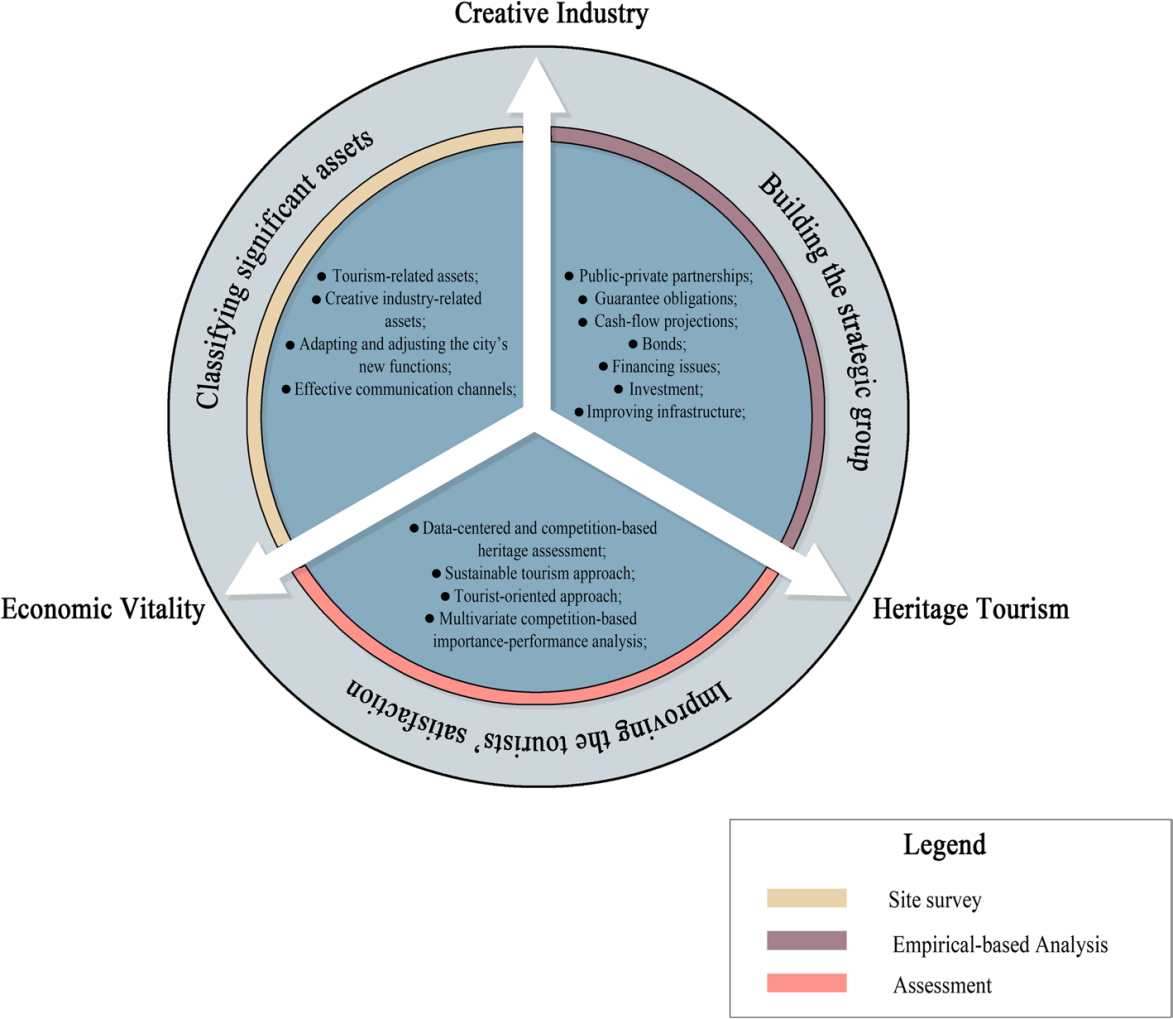
The creative industry’s contribution to Kulangsu Island's heritage tourism and economic vitality (Source: the author)

First, classifying the tourism-related and creative industry-related assets on Kulangsu Island based on the priority of heritage items is the first step. The unique qualities of a place, or its values, can be a large part of the tourism business and its key selling points, and they are also a benchmark for adapting and adjusting the city’s new functions to forge a new urban image and boost interests for all. Securing long-term sustainability for historic urban landscapes underpins the mutual interest of the creative industry and heritage tourism by triggering the economic vitality and potential value of urban assets in the long term. It is evident that sustainable heritage tourism closely aligns with the HUL approach, particularly concerning unique architecture, historical context, cultural diversity, and inclusiveness. Creative industries can also be a vehicle for positive change (Fig. 10): building culturally vibrant historic urban landscapes and becoming promising channels for connecting people's cultural needs with cultural production, which can generate both new visitor rates and economic opportunities to feed heritage conservation practices.

Second, a strategic group with heritage tourism, creative industry, and urban marketing and branding on Kulangsu Island should be built. Much experience has been provided by developed countries and successfully developed heritage sites in the fields of public‒private partnerships, guarantee obligations, cash-flow projections, bonds, and financing issues. Research points out that Southeast Asia’s dynamic economies, powered by a rising middle class, are strengthening inbound and outbound travel. The strategic group encompasses three preliminary requirements: first, public policies and economic incentives that support the development of creative industries and do the most to invest in infrastructure; second, government effectiveness in attracting official development assistance (ODA) and foreign direct investment (FDI); and third, the sustainability of heritage resources that facilitate the building of urban marketing and urban branding. Regarding the strategic group, the main driving force is the guiding role of the local authorities, the governmental ability to action, legally enforceable arrangements, the long-term implementation of policies, and requirements that are robust in the face of changing circumstances. Thus, the sustainability of the economy in the long term and its positive impact on all stakeholders and community groups are normally determined by new vitalities from culture, heritage, tourism, the creative industry, and urban marketing and branding. Furthermore, the value judgements and performance of public policy and conservation plans yield direct impacts on heritage tourism, the creative industry, and urban marketing and branding in terms of development prospects.

Third, the competitiveness of Kulangsu Island should be enhanced through data-driven and competition-based heritage assessments to increase visitor satisfaction. The historic urban landscapes are not just a choice of location but also represent an accumulation of customer pleasure with their goods, fresh energy, and a variety of services, as well as the ability to promote the location to others on the spur of the moment. At these sites, keen potential visitors are willing to pay for high-quality, authentic experiences. Let us review and reflect on the questions this study mentioned before: Imagine that you are walking on a historic commercial street on Kulangsu Island that does not have the uniqueness of place, and then compared it to the shophouse blocks in other areas of Fujian Province. Is it possible to evoke a feeling that different from the same type of streets in the old inner town of Xiamen, Zhangzhou, and Quanzhou? If there is a difference, what is it? And if there is no difference, why is that? Compared with the historic urban landscapes of Kulangsu Island in the past, what did we gain, and what did we lose? In the future, will there be more successful examples of conservation of historic urban landscapes or development of tourism projects (water parks, village travelling, coastal tourism activities) in heritage sites other than Kulangsu Island? Facing the current situation and examining the history, without effective product development, planning, marketing, management, interpretation, and expanding tourism activities on Kulangsu Island, the competitiveness of historic urban landscapes will not merely be decreased as time goes by but Kulangsu Island’s economic development prospects will also be weakened, and the days as a World Heritage site will be numbered, with perhaps only distinction being the UNESCO World Heritage site title. Here, a possible way to make a substantial effective contribution to policymakers and stakeholders regarding the most dynamic regional and international tour consumption market is by applying a sustainable tourism approach (McGehee et al. 2013), a tourist-oriented approach, and multivariate competition-based importance-performance analysis (MCIPA) (Guizzardi and Stacchini 2017) to tourists’ satisfaction statements.

####  Policy and planning: ensuring compatible development throughout all of the historic urban landscapes

##### (1) Fostering Public Cooperation and Industry Integration

On Kulangsu Island, the sustainability of historic urban landscapes is expected based on the good performance of public policies and conservation plans that foster resilient public cooperation and industry integration. In HUL practice, regional cooperation and integration should foster rapid and sustained social development and economic growth, which reduce the conflict and tension between different benefit clusters of heritage sites, improve the condition of infrastructure, reduce poverty, and build intraregional and extra regional collaboration mechanisms for mutually beneficial, broad-based, and inclusive growth. Theoretically, such a reciprocal relationship efficiently supports sustainability in Kulangsu Island’s historic urban landscapes directly and indirectly.

**Fig. 11:**
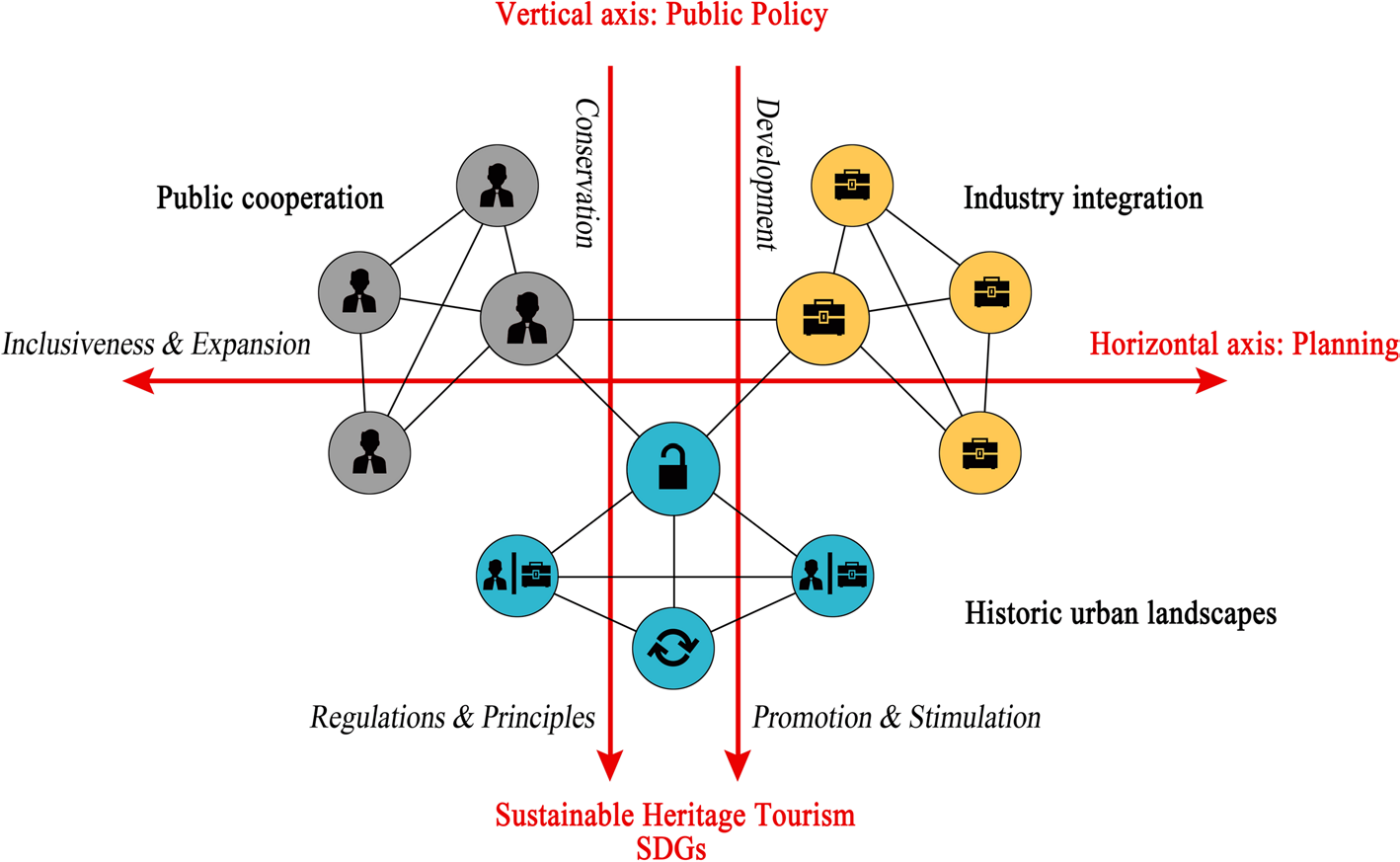
The vertical and horizontal framework for industry integration and public cooperation within certain rules and guidelines (Source: the author)

To sustainably achieve a balance between urban growth and the quality of historic urban landscapes, heritage administrators should envisage different fields of conservation actions occupying different areas of the development, investigation, and conservation triangle. Under suitable conditions, as shown in Fig. 11, effective public cooperation and industry integration are constructed vertically and integrated horizontally based on the hierarchically organised items and specialised principles of sustainability. Vertically constructed policies as a computer program can standardise and guide the correct solution to the sustainability need in pursuing sustainable macroscopically. Accordingly, horizontally integrated expansion of policies and plans can absorb and link the components of public cooperation and industry integration to meet sustainability needs concerning specific issues or potential sources of values that flexibly maximise public interests. Thus, when hundreds of activities occurred in Kulangsu Island’s historic urban landscapes, the policies and plans provided a vertical and horizontal framework for assessing whether programs, projects, and activities facilitated public cooperation and industry integration within specific regulations and principles. These programs, projects, and activities are related to an extensive exploration from basic built heritage research to tourism product planning, from assessment, testing, and preparation to cooperation, integration, marketing, and branding. Each such activity must be conducted with the guidance of the vertical and horizontal framework within specific regulations and principles.

##### (2) Fostering Economic Growth and Cultural Inclusion concerning Kulangsu Island’s historic urban landscapes

The level at which economic growth and cultural inclusion of a heritage site affect the local benefit output is well discussed in previous studies, which depend on countries’ structural characteristics, exposure to global markets, and policy design. Based on the culture-led and policy-led regeneration of Kulangsu Island’s historic urban landscapes, the historic urban landscapes are just a medium or space where the activity takes place, policies and plans are legal frameworks, and growth and inclusion are driving forces that connect all the potential elements. Additionally, strengthening governance and institutional capacity is important. Good governance and effective public management are essential for country and sector development (Asian Development Bank 2019). As reported in the Asian Development Bank’s Asia 2050 Report, better governance across a range of dimensions and Asia and the Pacific will determine whether a developing member country (DMC) can accelerate development and inclusive growth.

On Kulangsu Island, the challenges of growth, job creation, raising productivity levels, heritage assets, and inclusion are closely interlinked. The positive relationship between policies and plans and growth and inclusion is its competitiveness, sustainability, and transversality, which formulates economic output and inclusion. Economic growth focuses on opportunities and productivity, and cultural inclusion focuses on social justice, social cohesion, and community participation. In this light, policies and holistic planning should support strong economic growth, stability, and inclusive considerations on Kulangsu Island, which are prerequisites for the remaining sustainability of historic urban landscapes. In addition, the International Monetary Fund (2017) notes that although sustained growth is a precondition to support higher living standards and job creation, policies should also allow for the broad sharing of growth without affecting economic efficiency. When policies and plans enhance competitiveness and facilitate economies’ access to regional markets, a positive cycle will be formulated, and strong incentives and economic follow-up support will be generated for urban heritage assets.

The degree of cultural inclusion determines the quality of economic growth, which incorporates a spectrum of policies and holistic planning involving macro sustainable frameworks, from narrowest to most encompassing, from the monument site to the large-scale old streets and blocks, indirectly resulting in high quality and good performance of Kulangsu Island’s historic urban landscapes. It is not only a society’s culture that matters but also more general factors conducive to entrepreneurial activity (Beugelsdijk 2007). From a certain angle, economic growth and cultural inclusion have become something of a buzzword in sustainable development to enhance heritage-based competitiveness and productivity with the status of Kulangsu Island’s World Heritage site, yet each section requires adequate policy and planning response to support culturally relevant advantages that cannot be easily replicated.

##### (3) Stages in Identifying and Executing Interventions

**Fig. 12:**
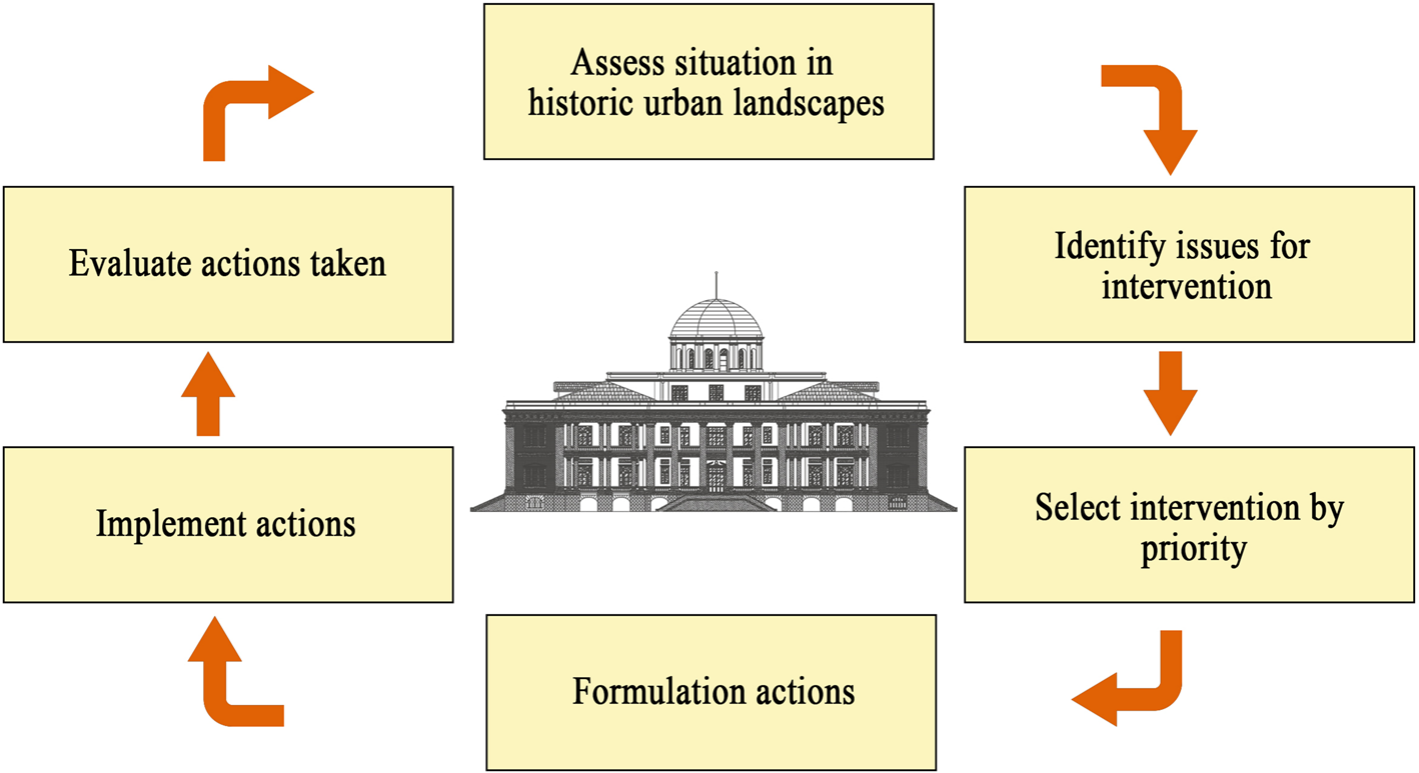
Each stage is outlined briefly in conducting policy-based and planning-based interventions (Source: the author)

On Kulangsu Island, the current policy and conservation plan concerning the sustainability of historic urban landscapes are only vague ideas, identifying and executing interventions much less than expected. The intervention needs a staged process from identification and design to execution. As shown in Fig. 12, a six-stage identifying and executing intervention periodically feeds into the future assessment of historic urban landscapes. Thus, based on the well-designed policy and structural plans and incorporating existing goals and targets, a virtuous circle is presented, which operates continuously, is reasonable, and responsive.

First, assessing situations in historic urban landscapes on Kulangsu Island involves a systematic process of analysis and prediction of the current situation concerning sustainability. The assessment will follow both bottom-up and top-down methods in Kulangsu Island’s overall urbanscape. The assessment comprises three parts: value assessment, economic assessment, and risk assessment. The value assessment involves the OUV of historic urban landscapes, related to both culture and heritage, and the prioritised historic buildings for intervention planning according to the classification of the latest offical heritage management measures of Kulangsu Island. Economic assessment refers to conducting a holistic assessment of existing economic development from the small business of private sectors to heritage tourism according to the opinions of residents, stakeholders, marketing specialists, heritage conservation specialists, and decision-makers. Risk assessment includes two processes: risk discovery processes and consultation processes. These two processes clarify the existing or potential risks.

Second, issues for intervention are identified through empirical evidence and data analysis. The purpose of this step is to extract complex and ever-growing issues in historic urban landscapes based on situation assessment and reasonable judgement. It encompasses four facets: (1) evaluating risks within the setting, (2) refining the issues, (3) analysing the issues, and (4) prioritising the issues for selecting further appropriate interventions. Essentially, the purpose of this step is to simplify the complexity of the process and create a priority setting concerning sustainability and historic urban landscapes.

Third, interventions were selected by priority after confirming the issues. This step covers the priorities of the government-identified projects or accelerating programs and related indicators regarding resourcing, time sequencing, and achievability. Researchers suggest the use of the multicriteria decision-making model (MCDM) and multicriteria decision analysis (MCDA) to evaluate the comprehensive impact of factors on matching the targeted issues, establish measurable criteria, and then carry out the selection of the intervention. In addition, big data analysis can support MCDA when confronted with confusion when selecting interventions by priority.

Fourth, a set of actions should be formulated to address the priorities in Kulangsu Island’s historic urban landscapes in a timely manner. Collaboration, peer outreach, and cooperation mechanisms are needed among different clusters, as this study discussed before, which includes residents, stakeholders, policymakers, marketing experts, and professionals training in different areas. Such broad collaboration serves as an important opportunity for communicating and exchanging information on action formulation.

Fifth, in pursuing successful targeted interventions, the action should be applied to heritage sites in a timely and effective manner to avoid diminishing returns due to time delay. Continuous and workable process management is needed for effective supervision, risk reduction, and conflict handling.

Sixth, in addition to monitoring and reporting during the enforcement process, an evaluation and review should be conducted at the end of the intervention. This would include a variety of metrics for assessing outputs, effectiveness, and value creation. Careful observation and analysis of the intervention enable the policies and plans to be updated or the procedure to be improved to produce a new plan to accommodate changing conditions and continuously minimise the possible negative effects of unanticipated new scenarios in historic urban landscapes.

## Conclusion

This study focused on discussions of the equilibrium model on Kulangsu Island, which covers an analysis combining public policies and conservation plans under a sustainable heritage tourism milieu. The equilibrium model of the HUL Recommendation is a dynamic framework that integrates social, economic, environmental, and cultural concerns into a holistic collaborative framework under a sustainable heritage tourism milieu. In line with the requirements of the historic urban landscape approach and general principles in support of sustainable urban heritage management promoted by UNESCO, the study points out the peculiarities and potential of the equilibrium mode in solving the current challenges of historic urban landscapes under the heritage tourism milieu. Finding ways of linking conservation, development, heritage tourism, and different interests groups to a holistic framework can stimulate effective means and management mechanisms for the complicated and changeable issues of sustainable heritage tourism.

Based on these findings, there is a range of questions for further study that could be explored:

First, in the aspect of social sustainability, the following two questions still need further discussion during sustainable heritage tourism development: how should development and competitiveness be aligned with respect for rights and the needs of inhabitants on Kulangsu Island together with highlighting urban heritage assets as public interest? How can social cohesion be ensured by the safeguarding of historic urban landscapes and inhabitants’ traditions and responding to the need for sociocultural acculturation of different generations? These questions are related to general political, cultural, and social conditions. Top-level policy design and an open attitude are crucial to respond to these questions.

Second, in terms of economic sustainability, further investigations that combine sustainable heritage tourism with the creative industry are needed on Kulangsu Island to adjust the industrial structure and economic vitality of the heritage sites. The question of how to use the HUL approach to create cross-cultural interactions between residents and outsiders is worth considering more. Fostering film production, advertising, publishing, exhibitions, art education, etc., on Kulangsu Island could bring new vigour and vitality into the urban space, and to some extent, it could be a way to boost the local tourism economy. This question involves investment attraction and long-term planning of heritage sites, and how to adjust the city’s development directions step by step needs to be discussed in detail in the future.

Third, in terms of public policy and conservation plans, further discussion is needed to study public policy and conservation plans. Specifically, this study raises three questions for the future: how can public policy and conservation plans be successfully carried out with appropriate intervention and means regarding sustainable heritage tourism? How do public policy and conservation plans address the need for linking policies, techniques, people, culture, the environment, and the economy synergistically? How do public policy and conservation plans contribute to better sustainable modes of Kulangsu Island’s multicultural living heritage city planning? How can infill buildings be controlled in terms of design with context and urban fabrics without losing the appropriate development opportunities in sensitive places of Kulangsu Island? Additionally, more investigation is required to determine the relationship between diversity in the policymaking process and the sustainability of the conservation strategy when heritage tourist products are proposed. Therefore, to answer these questions, further study is needed, and predictably, it is still a challenge of exploration at national, state, and local government levels and more positive contributions are needed from marketing experts, policymakers, residents, stakeholders, architects, artists, preservationists, strategic partners, and developers.

## Data Availability

Not applicable.

## Abbreviations

DMC: Developing member country
FDI: Foreign direct investment
HUL: Historic Urban Landscape
MCDA: Multicriteria decision analysis
MCDM: Multicriteria decision-making model
MCIPA: Multivariate competition-based importance-performance analysis
ODA: Official development assistance
OUV: Outstanding Universal Value
SDG: Sustainable Development Goals
